# The Antioxidant Capacity In Vitro and In Vivo of Polysaccharides From *Bergenia emeiensis*

**DOI:** 10.3390/ijms21207456

**Published:** 2020-10-09

**Authors:** Chen Zeng, Shiling Feng

**Affiliations:** College of Life Science, Sichuan Agricultural University, Ya’an 625014, China; zc15183819422@126.com

**Keywords:** *Bergenia emeiensis*, polysaccharides, antioxidant ability, *C. elegans*

## Abstract

Polysaccharides from *Bergenia emeiensis* (PBE) showed a robust antioxidant ability on scavenging free radicals in vitro. However, the further antioxidant potential in cell level and in vivo was still unknown. Therefore, in this present study, the protective effect of PBE on human cervical carcinoma cell (Hela) cells and *Caenorhabditis elegans* against oxidative stress was evaluated. The results showed PBE could reduce the reactive oxygen species (ROS) level in Hela cells and promote the mitochondrial membrane potential. Then, the cell apoptosis was reduced. Moreover, PBE could enhance the survival of *C. elegans* under thermal stress to 13.44%, and significantly reduce the ROS level, which was connected with the overexpression of *sod-3* and the increased nuclear localization of *daf-16* transcription factor. Therefore, PBE exhibited a strong antioxidant capacity in the cellular level and for a whole organism. Thus, polysaccharides from *B. emeiensis* have natural potential to be a safe antioxidant.

## 1. Introduction

As people pay more attention to health, free radicals, and other health hazards gradually move towards the focus of researches. Free radical is the general name of a series of atoms, atomic groups, or molecules in a special state containing unpaired electrons generated in the process of biochemical reactions in the body [1,2,3]. In the process of transforming O_2_ into H_2_O, a lot of reactive oxygen species (ROS) will be produced, such as superoxide anion free radicals, hydroxyl radicals, and hydrogen peroxide [4]. When the balance between production and scavenging of radicals is disrupted, excessive free radicals will lead to tissue damage, including DNA break, damage to biofilm structure, then even aging and cancer [5,6,7].

Antioxidants are a kind of substance that can effectively prevent or delay oxidation. Plants are the source of natural antioxidants with the characteristics of low toxicity and high efficiency. Polysaccharides from plants exhibit excellent antioxidant activity by effectively scavenging free radicals and increasing the activity of antioxidant enzymes [8,9,10]. Polysaccharides from four *Auriculariales fungus* could eliminate 1,1-diphenyl-2-picrylhydrazyl (DPPH) radicals significantly [11]. Five polysaccharides from *Inonotus obliquus* also showed a strong scavenging ability on radicals [12].

As an important model organism, *Caenorhabditis elegans* is widely used in drug screening for its fast reproduction speed, short life-cycle, easy observation, and the high homology of the immune and aging intervention regulation signal pathway to mammals [13]. Wang et al. found that *Angelica sinensis* protein hydrolysate could exert antioxidant activity and prolong the lifespan of *C. elegans* [14]. Moy et al. tested 6000 compounds and 1136 natural product extracts by using *C. elegans* infected by conditional pathogen *Enterococcus faecalis*, to uncover anti-infective molecular drugs [15]. Zhang et al. used *C. elegans* to assess the anti-microbial and anti-aging effect of the polysaccharide from *Sophora moorcroftiana* seeds [16]. Fang et al. used *C. elegans* to demonstrate that *Auricularia auricular* polysaccharides possessed a potential antioxidant activity [17].

*Bergenia emeiensis* C.Y.Wu, a plant of the genus Saxifragaceae, is a special species of China, natively distributed at Sichuan and growing in stone gaps at an altitude of 1300–1500 m [18]. In the area of mountain Emei, *B. emeiensis* was widely applied as folk medicine. However, there are few studies on the pharmacological activity and chemical compositions on *B. emeiensis*. The plants of *Bergenia* have been proved to exhibit good antioxidant activity [19]. In our previous work, the polysaccharides from *B. emeiensis* (PBE) could greatly scavenge DPPH and 3-ethylbenzothiazoline-6-sulfonic acid (ABTS) radicals [20]. However, the scavenging ability on radicals in vitro alone cannot comprehensively evaluate the antioxidant capacity of PBE. Consequently, further research needs to be implemented. Therefore, the protective effects of PBE on Hela cells and *C. elegans* were evaluated, so as to provide a theoretical basis for better utilization of *Bergenia emeiensis*.

## 2. Results

### 2.1. PBE Could Enhance the Cell Viability under Oxidative Stress

**Figure 1:**
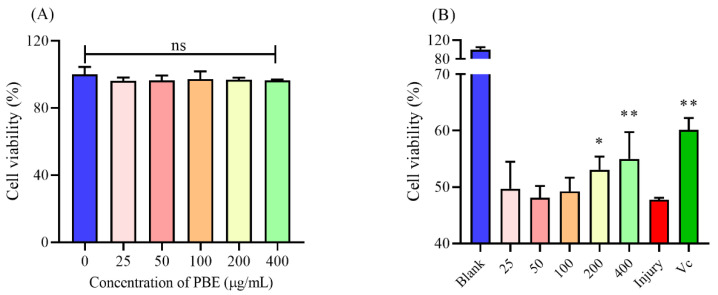
The effect of polysaccharides from *Bergenia emeiensis* (PBE) on (**A**) the cell viability of Hela cells for 24 h treatment and (**B**) the protection on cells against H_2_O_2_. Notes: * means *p* < 0.05 and ** means *p* < 0.01. Injury means the group treated only with H_2_O_2_.

Since PBE showed a strong antioxidant ability on scavenging DPPH free radicals, the inner effect on cell level needed further investigation. As shown in Figure 1A, in different concentrations of PBE on Hela cells for 24 h, the cell viability was not significantly changed (*p* > 0.05). Therefore, PBE showed no toxicity on cell proliferation, indicating PBE could be a naturally safe drug. Then, under the condition of H_2_O_2_, the cell viability was sharply decreased (Figure 1B). When the Hela cells were pretreated with PBE (25–100 μg/mL) for 24 h, the cell viability was not enhanced. A higher concentration of PBE (200–400 μg/mL) could obviously promote the cell survival rate under oxidative stress compared with the injury group. Therefore, like other natural polysaccharides, PBE also showed a protection effect on the cellular level. Gao et al. found that polysaccharides from angelica and garlic could improve cell survival [21].

### 2.2. PBE Could Reduce ROS Level

**Figure 2:**
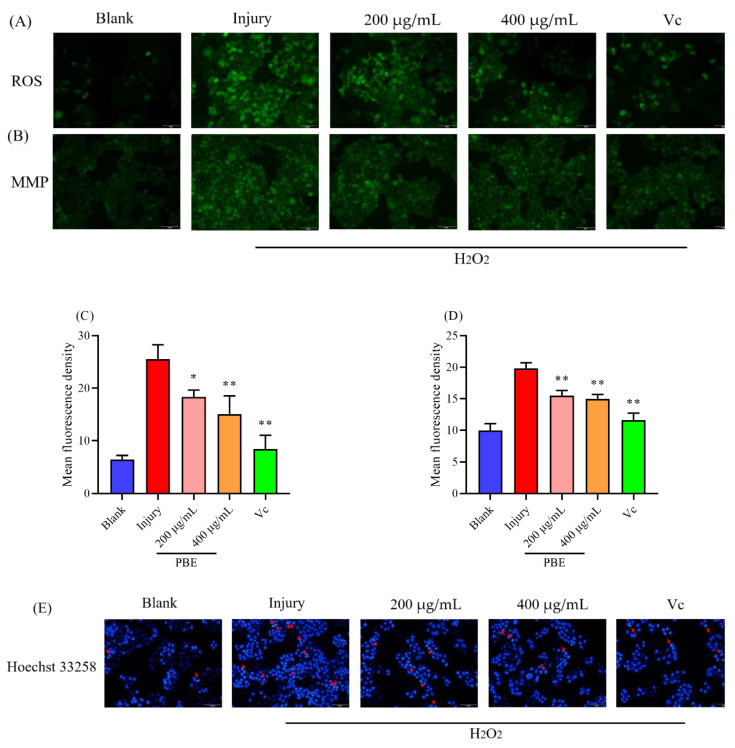
The effect of PBE on Hela cells under oxidative condition. (**A**) the reactive oxygen species (ROS) and (**B**) mitochondrial membrane potential (MMP) dying images under a fluorescence microscope. In addition, the changes in (**C**) the content of ROS and (**D**) mitochondrial membrane potential, and (**E**) the cell apoptosis in each group are shown. Notes: * means *p* < 0.05 and ** means *p* < 0.01. Red arrows mean the apoptotic bodies and injury means the group treated only with H_2_O_2_. The red bar is 50 μm.

H_2_O_2_ was a trigger for inner reactive oxygen species (ROS), such as ·OH, O_2_^−^ [22]. Under the H_2_O_2_ stress, the ROS level could be accumulated increasingly (Figure 2A), ascorbic acid (Vc) as a natural antioxidant could reduce the ROS level. Furthermore, PBE could decrease the ROS level in Hela cells, among which, 400 μg/mL of PBE showed a substantial effect on lowering ROS level to 40.89% (Figure 2C). A high level of ROS could lead to cell damage such as nucleic acid oxidation, lipid oxidation and protein oxidation, ultimately inducing cell death [23]. Astragalus polysaccharides could lower ROS of human umbilical vein endothelial cells under acute oxidative stress [24]. *Ziziphus jujuba* var. *spinosa* seeds polysaccharides also could enhance cell viability by reducing the leaping ROS [25]. Therefore, PBE might protect Hela cells by lowering the increasing ROS level.

### 2.3. PBE Could Improve the MMP Then Reduce Cell Apoptosis

In the previous reports, the content of ROS was associated with cell death. A high level of ROS could lead to cell apoptosis by damaging the structure of the mitochondrial membrane, thus causing a lower mitochondrial membrane potential [26]. When the integrity of the mitochondrial membrane was broken, rhodamine 123 was released from mitochondria, consequently showing a strong green fluorescence. As shown in Figure 2B, when the HeLa cells were exposed to H_2_O_2_, the green fluorescence was stronger than the blank group, meaning the mitochondrial membrane potential (MMP) was sharply decreased. However, when the cells were pretreated with Vc and PBE for 24 h, the MMP could be promoted (Figure 2D). A low MMP could release cytochrome C then induce cell apoptosis. In the presence of H_2_O_2_, the cells suffered more cell apoptosis (Figure 2E). Since PBE could promote the MMP, less cell apoptosis appeared in the trial groups.

### 2.4. PBE Could Improve the Survival of C. elegans under Hot Stress

**Figure 3:**
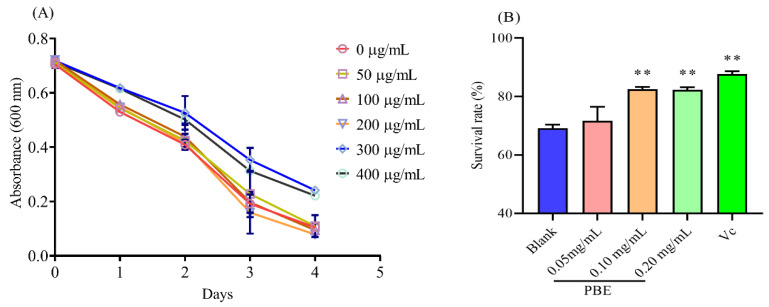
The effect of PBE on *C. elegans*. (**A**) The changes in absorbance in food clearance assay and (**B**) the protection of PBE on *C. elegans* under thermal stress. Notes: ** means *p* < 0.01.

Only in vitro assays could not comprehensively evaluate the antioxidant capacity of PBE. Therefore, *C. elegans* was adopted as the model organism to assess the antioxidant potential of PBE in vivo. The effect of different concentrations of PBE on the consumption of worms is shown in Figure 3A. Under limited conditions, the OP50 was consumed by the worms daily, reflecting the reduction of absorbance. Compared with the blank group, 300 and 400 μg/mL of PBE negatively affected the intake by the worms (Figure 3A). Hence, 0–200 μg/mL of PBE was used for further investigations. *C. elegans* was cultured at 20 °C and when the temperature was shifted to 35 °C, the worms were in a stress condition, leading to their death. An amount of 50 μg/mL of PBE could not improve the survival of worms, while 100 and 200 μg/mL of PBE could significantly enhance the hot resistance. The survival of worms pretreated with 100 and 200 μg/mL of PBE increased to 13.44% and 13.26%, respectively (Figure 3B). Hence, PBE could protect *C. elegans* in vivo against the stress environment.

### 2.5. PBE Could Reduce the ROS Level in C. elegans

**Figure 4:**
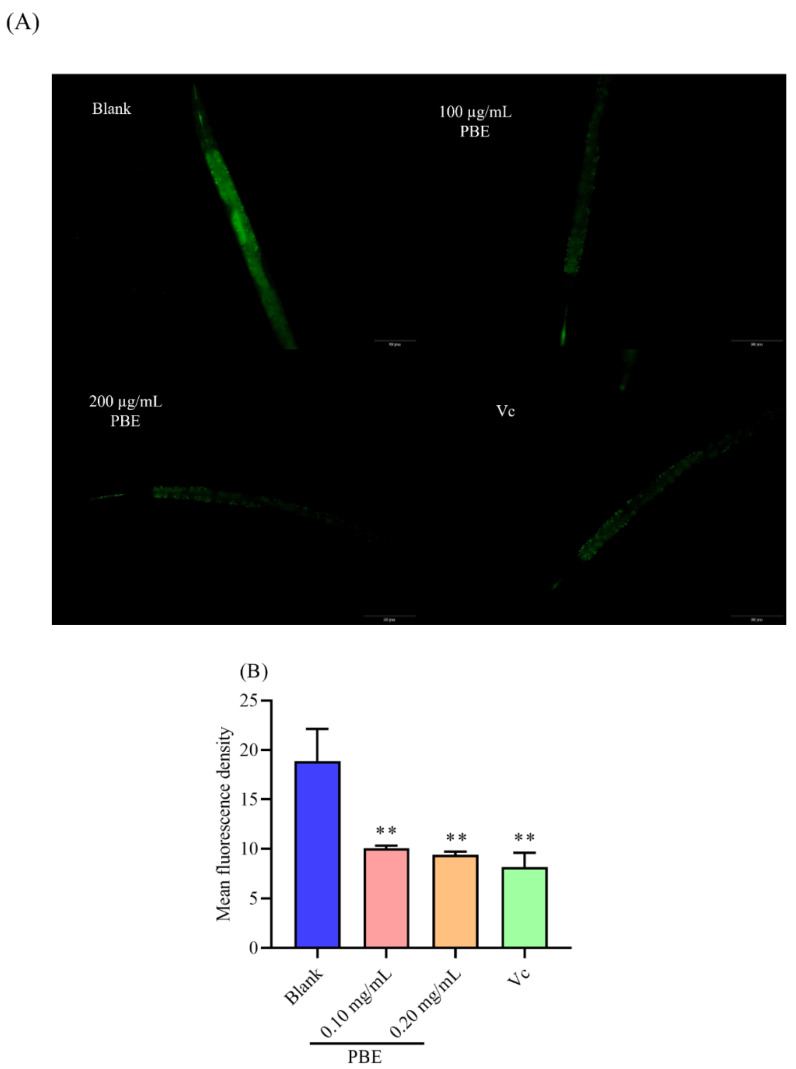
(**A**) The ROS level in the *C. elegans* under a fluorescence microscope and (**B**) the effect of PBE on the content of ROS under thermal stress. Notes: ** means *p* < 0.01. The red bar is 50 μm.

Under thermal stress, *C. elegans* suffered oxidative damage, leading to a high level of ROS [27]. The balance between generation and clearance of ROS was disturbed, then more free radicals could damage the organism [28]. As shown in Figure 4A, the ROS level was pretty high in the blank group. After pretreatment with PBE and Vc, the ROS content was obviously decreased to 46.78% and 50.17%, respectively (Figure 4B). Pumpkin polysaccharides were proved to lower the ROS in *C. elegans* under acute oxidative and thermal stress [29]. In addition, other natural polysaccharides could also reduce the increasing ROS level [30].

### 2.6. PBE Could Activate daf-16 Transcription Factor

**Figure 5:**
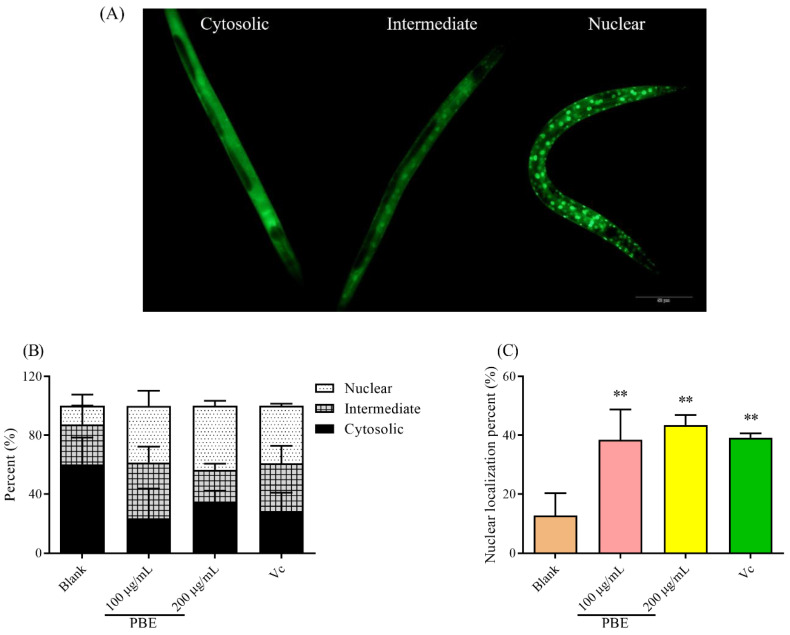
(**A**) The localization of transcription factor of *daf-16* in *C. elegans*. Pretreated with PBE, (**B**) the localization of *daf-16* and (**C**) the percentage of nuclear localization. Notes: ** means *p* < 0.01. The red bar is 50 μm.

*Daf-16* was the central regulation on the resistance to the stress environment. Since PBE could improve the resistant ability of *C. elegans* under thermal stress, the localization of transcription factor *daf-16* was observed. When the *daf-16* was not turned into the nucleus, the whole body of *C. elegans* showed a diffused green fluorescence (Figure 5A), while when the *daf-16* was activated, the transcription was turned into the nucleus showing a bright spot. As shown in Figure 5B, the cytosolic localization was reduced after being treated with PBE, while *daf-16* was turned more into the nucleus. PBE could increase the percentage of nuclear localization of *daf-16* in *C. elegans* from 12.70% to 38.43% and 43.46%, respectively (Figure 5C). Hence, PBE promoted the resistance of *C. elegans* by activating the *daf-16* transcription factor.

### 2.7. PBE Could Promote the Expression of sod-3

**Figure 6:**
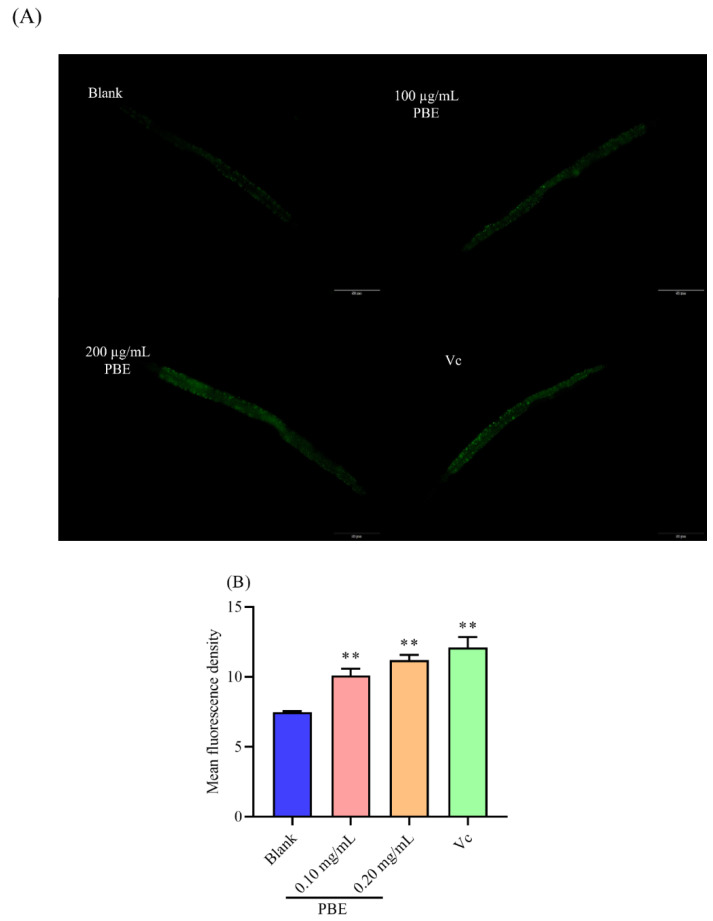
(**A**) The observation of *sod-3* in *C. elegans* under thermal stress and (**B**) the effect of PBE on the expression of *sod-3* in *C. elegans*. Notes: ** means *p* < 0.01. The red bar is 50 μm.

In the system of a whole organism, the antioxidant enzymes were the principal defense system against the increasing ROS level [31]. Antioxidant enzymes such as superoxide dismutase (SOD) were located downstream of *daf-16*. Since PBE could activate the *daf-16* transcript factor, a transgenic strain carrying *sod-3p*::GFP was applied to investigate the effect of PBE on the antioxidant system. As shown in Figure 6A, *C. elegans* pretreated with PBE for 48 h expressed more *sod-3*, compared with the blank group. In addition, 200 µg/mL PBE showed a strong effect by promoting the expression of *sod-3* to 50.07% (Figure 6B). Therefore, it could be concluded that PBE activated the transcription factor of *daf-16* then up-regulated the antioxidant enzymes such as *sod-3*, reflecting the boosting expression of *sod-3*, hence reducing the increasing ROS and enhancing the survival of *C. elegans* under thermal stress.

## 3. Discussion

Oxidative damage is one of the most common injuries in the body caused by the metabolic activities of life [32]. When some biological macromolecules such as protein and nucleic acid are damaged, it leads to tissue injury, and even aging of the whole body [33]. A variety of diseases are also highly associated with oxidative damage, such as coronary heart disease, hypertension, stroke [34], Alzheimer’s disease [35], cancer [36] and diabetes [37]. The oxidative damage of the body is mainly caused by the overproduction of free radicals. Therefore, detecting the scavenging of free radicals has become an important index to evaluate the antioxidant capacity of drugs.

Polysaccharides are biological macromolecules with a wide range of biological activities in the pharmaceutical industry due to their robust antioxidant properties [38]. In our previous studies, we confirmed that *PBE* had a strong antioxidant capacity for scavenging DPPH and ABTS free radicals in vitro [20]. Wang et al. found that ginger polysaccharides exhibited a good scavenging activity on DPPH radicals [39]. Chen et al. found that *Momordica charantia* polysaccharides could clear superoxide anion, hydroxyl and DPPH radicals well [40]. Garlic polysaccharides were also proved to exert a good scavenging activity on radicals [41].

However, the simple test of metal ion chelation and free radical scavenging alone cannot simulate the antioxidant activity of drugs in the real biological environment well, and the cell model has been recognized as a more accurate and real method to assess the bioactivity of natural products by the main scientific community [42]. The oxidative damage of cells is mainly manifested in increase in reactive oxygen species, and increase in ROS will lead to decrease in mitochondrial membrane potential, leading to apoptosis [43]. As a free radical inducer, hydrogen peroxide can cause a sharp increase in free radicals in cells, thus damaging cells [44]. An et al. found *Cyclocarya paliurus* polysaccharides could improve the antioxidant capacity of RAW264.7 [45]. Sun et al. found that green tea polysaccharides could reduce the production of reactive oxygen species in human renal tubular epithelial cells (HK-2) and increase mitochondrial membrane potential, showing a strong antioxidant activity [46]. *Taraxacum mongolicum* polysaccharides also had a protective effect on oxidative damage of human normal liver cells (LO2) [47]. Like other natural polysaccharides, PBE could reduce the level of reactive oxygen species in cells and the damage of ROS to the mitochondrial membrane, thus alleviating the decrease in mitochondrial membrane potential and cell apoptosis, showing a protective effect.

*C. elegans* has been widely used in drug screening [48,49]. Compared with in vitro experiments, *C. elegans* can better reflect the effects of substances on organisms as a whole. Lublin et al. found that caffeine, tannic acid and bacitracin could protect *C. elegans* CL2006 strain from Aβ induced toxicity [50]. He et al. used *C. elegans* CL2355 as an Alzheimer disease model to test the neuroprotective activities of seven new 2-arylvinylquinoline derivatives [51]. *C. elegans* is maintained at a low temperature. When the worms are shifted to a high temperature, the level of reactive oxygen species in *C. elegans* will increase, thus making the nematodes suffer oxidative stress. The transcription factor of *daf-16* in *C. elegans* is the main regulatory factor under stress conditions. When *daf-16* is turned into the nucleus, expression of the downstream target gene, including antioxidant enzymes, will be promoted, consequently relieving the oxidative damage [52,53]. In this study, PBE promoted the entry of *daf-16* transcription factor into the nucleus of *C. elegans*, and the expression of downstream gene *sod-3*. Therefore, PBE might decrease the level of ROS, and improve the survival rate of *C. elegans* under thermal stress via *daf-16* pathways. Fang et al. found that *Auricularia auricular* polysaccharides can up-regulate the expression of *daf-16* and enhance the resistance of *C. elegans* [17]. Zhang et al. revealed that *Lycium barbarum* polysaccharides could activate *daf-16* then enhance the antioxidant ability of *C. elegans* under thermal stress [54]. In addition, *skn-1* and other factors also play a vital role in enhancing the resistance of *C. elegans* in stress environments [55]. Polysaccharides from Auricularia auricular could activate *skn-1* to improve the survival of *C. elegans* under thermal conditions [17]. The *Cyclocarya paliurus* polysaccharide also enhanced the antioxidant ability of *C. elegans* through *skn-1* [56]. Since PBE could strengthen resistant ability in *C. elegans* through *daf-16*, other signal pathways, like *skn-1*, might be involved. Therefore, further investigations will be carried out to explore the antioxidant mechanism.

## 4. Materials and Methods

### 4.1. Materials and Reagents

*B. emeiensis* was picked from Mountain Emei, Sichuan, China, at the altitude of 1300–1500 m. The rhizomes of *B. emeiensis* were washed with distilled water to remove dirt and dried naturally. Then the rhizomes were grounded into fine powder sieving with a mesh of 60 and stored at 4 °C.

Dimethyl surtoxide (DMSO), ascorbic acid (Vc), sodium chloride, chloroform, magnesium sulfate, calcium chloride, and others were purchased from Kelong Chemical Factory (Chengdu, China). Reactive oxygen species (ROS) reagent, Hoechst 33258 was bought from Beyotime Biotechnology (Shanghai, China). 3-(4,5-dimethyl-2-thiazolyl)-2,5-diphenyl tetrazolium bromide (MTT) and agar powder were obtained from Beijing Solarbio Science and Technology Co., Ltd. (Beijing, China). Rhodamine 123, Fetal bovine serum (FBS), Dulbecco’s modified eagle medium (DMEM), phosphate buffered solution (0.0067 M, PBS) and trypsin were from Hyclone (Logan, UT, USA).

### 4.2. The Extraction and Purification of Polysaccharides from B. emeiensis (PBE)

As described in our previous report, the powder of *B. emeiensis* was mixed with distilled water and extracted under the optimal extraction conditions [20]. Then the extraction solution was concentrated with a rotary evaporator, and 4 times the volume of ethanol was added in. The precipitation was obtained after standing 24 h at 4 °C and dissolved in distilled water with 10% trichloroacetic acid. The solution was placed at 4 °C for 24 h, and the dirt was removed after centrifugation. The supernatant was dialyzed for 3 days, and the dialysate was deproteinized with the Savage method. Then the aqueous solution was lyophilized to gain the polysaccharides from *B. emeiensis* (PBE). The content of polysaccharides was more than 90%.

### 4.3. The Protection of PBE on Hela Cells against H_2_O_2_

#### 4.3.1. The Culture of Hela Cells

Hela cells were kindly provided from Stem Cell Bank, Chinese Academy of Science, Shanghai, China. Hela cells were maintained in DMEM containing 10% FBS at 37 °C in an atmosphere of 5% CO_2_. When the cells reached to about 80% of the flask, the cells were digested from the bottle and washed with PBS 3 times for the following assays.

#### 4.3.2. The Toxicity of PBE on Hela Cells

The cell viability was measured according to the MTT method described in the previous report [57]. The density of Hela cells was adjusted to 10^5^ cells/mL. Then a 90 μL cell suspension was seeded in a 96-well plate. After 6 h, 10 μL of the PBE sample solution (resolved in distilled water) was added into the plate for 24 h. Then all the solution was removed, and fresh medium and MTT (dissolved in PBS, 5 mg/mL) were added for 5 h at 37 °C. Subsequently, the solution was replaced with DMSO, and the plate was shaken for 1 h before the absorbance of each well was read at 570 nm. The experiments were repeated three times and there were five wells in each concentration. The cell viability was calculated according to the following formula:

Cell viability (%) = A1/A2

where A1 was the absorbance of cells treated with sample solution and A2 was the absorbance of cells treated with distilled water instead as a blank group.

#### 4.3.3. The Protective Effect of PBE on Hela Cells against H_2_O_2_

The cell suspension was adjusted to 10^5^ cells/mL and seeded into the 96-well plate. After 6 h incubation, different concentrations of PBE solution were transferred into each well. The Vc (10 μg/mL) was used as the positive control. In the injury and blank groups, in the same volumes of distilled water were added instead. After 24 h treatment, the solution was removed and H_2_O_2_ (final concentration of 4 mM) was added into the plate for 5 h. Subsequently, H_2_O_2_ was removed, and new medium as well as the MTT solution were transferred into the plate. Then the cell viability was determined according to the MTT method. The experiments were repeated three times and there were five wells in each concentration.

#### 4.3.4. Fluorescence Staining Assays

The cells were seeded into a 6-well plate for 12 h incubation. Then the cells were treated with PBE for another 24 h. Subsequently, the sample solution was replaced with H_2_O_2_. After 5 h exposure, the H_2_O_2_ solution was removed, and 4% paraformaldehyde was transferred into the plate to fix cells at 4 °C for 30 min. Then PBS was used to wash the cells 3 times, and ROS reagent (DCFH-DA, 2′,7′-Dichlorofluorescin diacetate), Hoechst 33258 and rhodamine 123 were added into the plate for 30 min, 25 min and 35 min, respectively. Finally, the ROS level, cell apoptosis and mitochondrial membrane potential (MMP) were observed under a fluorescence microscope. The experiments were repeated three times and each well was pictured randomly, and three pictures were randomly collected for each concentration.

### 4.4. The Protection of PBE on C. elegans against Oxidative Stress

#### 4.4.1. The Culture of *C. elegans*

Wild-type N2, TJ356 *daf-16*::gfp (zIs356 (p*DAF-16*::*DAF-16*-GFP; rol-6)), CF1553 (muIs84 [(pAD76) *sod-3*p::GFP + rol-6(su1006)]), *C. elegans* and *Escherichia coli* OP50 strain were obtained from the Caenorhabditis Genetics Center (CGC). The worms were cultured on nematode growth medium (NGM) with a layer of OP50 as the food source at 20 °C. Synchronized worms were obtained by the sodium hypochlorite method [58].

#### 4.4.2. Food Clearance Assay

The food clearance experiment was conducted to select non-toxic concentrations of PBE on *C. elegans* as previously described [59]. Briefly, PBE was dissolved in distilled water to different concentrations. Synchronized worms at the L4 stage were transferred into a 96-well plate at 20–30 worms/well. In additon, dead OP50 strains and PBE sample solutions were added into the plate. 5-fluorouracil (final concentration was 25 μM) was used to prevent worms from reproduction. The experiments were repeated three times and there were five wells in each concentration. The absorbance of the plate was read at 600 nm daily.

#### 4.4.3. Thermal Stress Assay

Synchronized worms were placed on the NGM plate with and without the presence of PBE for 48 h. Then, the worms were transferred to a new plate without PBE. Vc (10 μg/mL) was used as the positive control. Subsequently, the plate was placed at 35 °C for 5 h then put at 20 °C for 24 h. The survival of worms was counted. The experiments were repeated three times and there were at least 25 worms in each concentration.

#### 4.4.4. Determination of ROS level

Adult worms were placed on the NGM plate for 3 h to lay eggs. Then the worms were removed, and the plate was maintained at 20 °C until the synchronized worms grew to L4 stage. The nematodes were exposed to 35 °C for 1 h and washed from the plate with a liquid medium, and washed 7 times to remove OP50. Then ROS reagent was added into the liquid medium with the worms for 1 h in the dark. Afterwards, the worms were anesthetized with sodium azide (15 mM). The ROS level was observed with a fluorescence microscope. The experiments were repeated three times and there were at least 10 worms in each concentration.

#### 4.4.5. Visualization the Localization of *daf-16*

A transgenic strain TJ 356 carried a GFP on the transcription factor of *daf-16*, making the visualization of *daf-16* clearness. Briefly, the synchronized worms grew on the plate in the absence and presence of PBE to L4 stage. Then the worms were fixed on the glass slide with sodium azide. The sublocalization of *daf-16* was recorded by a fluorescence microscope as cytosolic, intermediate, and nuclear. The experiments were repeated three times and there were at least 15 worms in each concentration.

#### 4.4.6. Visualization of the Expression of *sod-3*

CF1553 strain carrying a *sod-3*::GFP was used to easily quantize the expression of *sod-3*. Hence, the transgenic worms were placed on the NGM plate for 48-h treatment. Then the plate was put at 35 °C for 2 h. In addition, sodium azide was used to anaesthetize the worms on a microscope slide. The expression of *sod-3* was observed with a fluorescence microscope. The experiments were repeated three times and there were at least 10 worms in each concentration.

### 4.5. Statistical Analysis

The data obtained in this work were analyzed with GraphPad Prism 6 (GraphPad Software, Inc., La Jolla, CA, USA). The relative fluorescence intensity was quantified with Image-J software (National Institutes of Health, Bethesda, MD, USA). All values were expressed as mean ± standard deviation (SD), and experiments were performed in triplicate. The data were analyzed with Analysis of Variance (ANOVA) and *p* < 0.05 level was considered to be significantly different.

## 5. Conclusions

In the present study, PBE could reduce the ROS level in Hela cells under H_2_O_2_ conditions and promote the mitochondrial membrane potential. Then, cell apoptosis decreased and the cell viability pretreated with PBE was improved. Therefore, PBE showed great protection on the cellular level against acute oxidative stress. Further investigations demonstrated PBE could also protect *C. elegans* under thermal stress showing an increase in survival to 13.44%. Moreover, PBE could help *daf-16* transcription factor move into nucleus, increasing the percentage of transfer from 12.70% to 43.46% (200 µg/mL of PBE). The expression of *sod-3* was increased, while the ROS level was sharply decreased. Therefore, PBE exhibited a strong antioxidant capacity in vivo through activating *daf-16* and enhancing the expression of antioxidant enzymes such as *sod-3*, reducing the ROS level. Overall, PBE was a potential natural antioxidant and could be applied in the pharmaceutical industry.
